# Effectiveness and renal safety of dupilumab in the treatment of moderate-to-severe atopic dermatitis patients with chronic kidney disease stages 4–5: a real-world cohort study in China

**DOI:** 10.3389/fmed.2026.1889589

**Published:** 2026-08-27

**Authors:** Gang Hu, Quanhong Zhang, Luoyao Yang, Yanglin Hu, Jinbo Chen

**Affiliations:** 1 School of Medicine, Jianghan University, Wuhan, China; 2 Department of Dermatology, Traditional Chinese and Western Medicine Hospital of Wuhan, Tongji Medical College, Huazhong University of Science and Technology, Wuhan, China; 3 Department of Dermatology, Wuhan No.1 Hospital, Wuhan, China; 4 Hubei Province & Key Laboratory of Skin Infection and Immunity, Wuhan No. 1 Hospital, Wuhan, China; 5 Department of Nephrology, Wuhan No.1 Hospital, Wuhan, China; 6 Department of Dermatovenereology, West China Hospital, Sichuan University, Chengdu, China

**Keywords:** atopic dermatitis, dupilumab, efficacy, real-world study, renal safety, severe renal failure

## Abstract

**Background:**

While dupilumab is widely used for atopic dermatitis (AD), its safety and effectiveness in patients with severe renal failure remain insufficiently studied.

**Objective:**

To evaluate the real-world effectiveness and renal safety of dupilumab in this population.

**Methods:**

This cohort study included 30 adult moderate-to-severe AD patients with Chronic Kidney Disease (CKD) stages 4–5. Patients were divided into a dupilumab-treated group (*n* = 17) and a control group (*n* = 13) receiving conventional therapy. Effectiveness was assessed using the Peak Pruritus Numerical Rating Scale and Investigator's Global Assessment. Renal safety was evaluated via eGFR and serum creatinine levels, analyzed using linear mixed-effects models.

**Results:**

The dupilumab group showed a numerically greater PP-NRS reduction at Day 30 (4.6 vs. 2.7 points) with a trend toward sustained improvement; however, the between-group difference did not reach statistical significance in the mixed-effects model (*P* = 0.109). IGA scores showed similar trends. Regarding renal safety, despite poorer baseline renal function in the dupilumab group, the degree of renal function decline was not statistically different from that in the control group. Sensitivity analysis excluding patients on dialysis did not alter this conclusion.

**Conclusion:**

Dupilumab was associated with a numerical trend toward greater improvement in pruritus compared with conventional therapy, and was not associated with statistically detectable acceleration of renal function decline in AD patients with CKD stages 4–5. These preliminary findings require confirmation in larger prospective studies.

## 1 Introduction

Atopic dermatitis (AD) is a chronic, pruritic, immune-mediated inflammatory skin disease characterized by a predominant Th2-type inflammatory response, and frequently associated with systemic inflammation. Dupilumab, a fully human IgG4 monoclonal antibody, has demonstrated substantial efficacy in patients with moderate-to-severe AD by targeting IL-4Rα and thereby inhibiting both IL-4 and IL-13 signaling pathways. Pharmacokinetically, dupilumab exhibits a relatively small volume of distribution, is eliminated slowly, and is primarily metabolized through non-renal pathways, including proteolytic degradation and target-mediated drug disposition. According to the prescribing information, no dosage adjustment is necessary for patients with mild or moderate renal impairment. However, data on the use of dupilumab in individuals with severe renal impairment remain scarce, and caution is warranted. Although its pharmacokinetic profile suggests minimal reliance on renal clearance (1), clinical evidence in patients with advanced chronic kidney disease [CKD stages 4–5, estimated glomerular filtration rate (eGFR) ≤ 29 mL/min] is limited. Because patients with severe renal dysfunction are predisposed to altered drug metabolism and a heightened risk of adverse drug events (2). Therefore, this study aims to evaluate the real-world application effectiveness and renal safety of dupilumab in patients with CKD stages 4–5 through a retrospective analysis, thereby addressing an existing gap in clinical evidence.

## 2 Methods

### 2.1 Study design and patient grouping

This study retrospectively collected data from adult patients with moderate-to-severe AD who were treated in Wuhan No.1 Hospital between January 2020 and August 2024 and divided them into two groups according to the treatment regimen.

Dupilumab group: AD patients with CKD stages 4–5 (eGFR ≤29 mL/min) receiving dupilumab treatment;

Control group: AD patients with CKD stages 4–5 receiving conventional therapy.

### 2.2 Inclusion and exclusion criteria

Inclusion criteria: (1) Age ≥18 years; (2) Meeting AD diagnostic criteria (Hanifin & Rajka. Disease duration was not a mandatory inclusion criterion but was collected as a descriptive baseline characteristic. To minimize misclassification with CKD-associated pruritus, eligible patients were required to have inflammatory eczematous lesions with characteristic primary lesion morphology and distribution, including erythema, papules, lichenification or flexural eczematous lesions; patients who presented only with generalized pruritus without inflammatory skin background, or with purely secondary scratching-related lesions such as simple excoriations, erosions, or prurigo nodularis, were excluded; (3) Possessing relatively complete clinical data (at least 2 sets of IGA scores and renal function data); (4) Clinical diagnosis of CKD stages 4–5 (eGFR ≤29 mL/min); (5) Baseline IGA score ≥3 (moderate-to-severe AD was defined as IGA ≥3 on the validated 5-point scale, consistent with international guidelines recommending systemic therapy for patients with IGA ≥3) (3); (6) Voluntary choice of treatment with either dupilumab or conventional therapy.

Exclusion criteria: (1) Patients whose renal impairment was caused by acute kidney injury (AKI); (2) Pregnant or lactating women; (3) Patients with advanced cancer; (4) Patients who did not complete effectiveness and safety evaluations; (5) Patients concurrently using nephrotoxic medications during treatment that could affect renal function assessment.

### 2.3 Grouping criteria and treatment regimen

A total of 30 patients were included in this study, with 17 in the dupilumab group and 13 in the control group. Grouping criteria were as follows: patients in the dupilumab group received dupilumab treatment for at least 16 weeks, while those in the control group did not receive dupilumab. There were no statistically significant differences between the two groups in terms of gender, age, baseline IGA, PP-NRS, eGFR, serum creatinine (Cr) (all *P* > 0.05), but given the inherent biases of observational studies, baseline comparability should be interpreted with caution; furthermore, there were significant differences between the two groups in dialysis status (*P* = 0.024) and proportion of topical corticosteroid use (*P* = 0.007), suggesting potential confounders (see Table 1).

**Table 1:** Demographics and baseline disease characteristics.

Label	Total (*N* = 30)	Dupilumab group	Control Group	*P* value
Number of Subjects	30	17	13	
Age, mean ± SD, years	73.9 ± 10.9	73.5 ± 10.59	74.5 ± 12.32	0.817^a^
Gender (Male/Female)	26/4	15/2	11/2	1.000^b^
Comorbid Hypertension, *n* (%)	24 (80.0%)	13 (76.5%)	11 (84.6%)	0.672^b^
Comorbid Diabetes, *n* (%)	12 (40.0%)	5 (29.4%)	7 (53.8%)	0.264^b^
Comorbid Coronary Heart Disease, *n* (%)	10 (33.3%)	4 (23.5%)	6 (46.2%)	0.255^b^
Average AD Duration (months), median (IQR)	11.2 (6.2–15.9)	11.5 (6.5–16.6)	10.7 (5.0–13.4)	0.568^c^
Average CKD Duration (months), median (IQR)	15.5 (7.3–32.0)	16.5 (9.5–41.5)	11.9 (6.2–19.8)	0.278^c^
Oral Corticosteroid Use, *n* (%)	8 (26.7%)	4 (23.5%)	4 (30.8%)	0.699^b^
Maintenance Dialysis, *n* (%)	6 (20.0%)	6 (35.3%)	0 (0%)	0.024^b^
RAAS Inhibitors/Diuretics Use, *n* (%)	25 (83.3%)	15 (88.2%)	10 (76.9%)	0.628^b^
Baseline IGA, mean ± SD	3.97 ± 0.57	3.94 ± 0.66	4.0 ± 0.43	0.766^a^
Baseline NRS, mean ± SD	8.28 ± 0.84	8.06 ± 0.90	8.58 ± 0.67	0.080^a^
Baseline GFR, median (IQR)	20.41 (9.17–28.76)	18.05 (6.57–23.78)	20.53 (14.88–33.63)	0.380^c^
Baseline CR, median (IQR)	243 (174–508)	258.0 (174.0–508.0)	224.5 (167.5–321.5)	0.380^c^
Baseline Total IgE (IU/mL), median (IQR)	–	173.0 (41.4–382.8)	944.0 (137.5–1,412.5)	0.157^c^
Baseline Eosinophil Count (×10^9^/L), median (IQR)	–	0.24 (0.10–1.00)	0.80 (0.17–1.11)	0.298^c^
Topical Corticosteroid Use, *n* (%)	19 (63.3%)	7 (41.2%)	12 (92.3%)	0.007^b^
Comorbid Allergic Rhinitis, *n* (%)	7 (23.3%)	5 (29.4%)	2 (15.4%)	0.425^b^
Comorbid Asthma, *n* (%)	1 (3.3%)	1 (5.9%)	0 (0%)	1.000^b^
Oral Antihistamine Use, *n* (%)	29 (96.7%)	16 (94.1%)	13 (100%)	1.000^b^
Adverse Reactions (e.g., Injection Site)	0	0	0	

IGA, investigator's global assessment; IQR, interquartile range; n, number; PP-NRS, peak pruritus numeric rating scale; SD, standard deviation.

a Independent Samples *t*-test.

b Fisher's Exact Test.

c Mann–Whitney *U*-test.

Treatment regimen: Patients in the dupilumab group were administered dupilumab with an initial dose of 600 mg, followed by 300 mg every 2 weeks via subcutaneous injection. During treatment, most patients received concomitant therapy with topical corticosteroid ointments or topical calcineurin inhibitors (TCIs), antihistamines, and emollients. A small number of patients (Table 1) received low-dose oral corticosteroids (prednisone 5 mg/day) as rescue therapy, with a maximum duration of treatment not exceeding one month. The control group received a similar basic treatment regimen—topical corticosteroids, TCIs, antihistamines, and emollients—except that dupilumab was not included.

### 2.4 Data collection

Data collection included demographic and clinical information such as age, gender, comorbid chronic diseases (hypertension, diabetes, coronary heart disease), and the duration of AD, together with changes in IGA and pruritus NRS scores during treatment. Records of patients' concomitant oral and topical medications during treatment were collected, including the use of corticosteroids, antihistamines, topical corticosteroid ointments, topical calcineurin inhibitors (TCIs), and emollients. Dupilumab-related adverse reactions (injection site reactions, conjunctivitis, and other herpes simplex virus infections) were recorded. Changes in renal function during treatment (based on eGFR and Cr), as well as any treatment-related adverse events, were also documented. Outcome measures included the proportion of patients achieving Investigator's Global Assessment (IGA) 0/1, representing almost clear skin, recorded as a secondary effectiveness outcome during follow-up. EASI was not included as an effectiveness endpoint because it was not systematically recorded in routine electronic medical records and therefore could not be reliably extracted retrospectively.

### 2.5 Statistical analysis

Statistical analyses were performed using SPSS 26.0 and R (v4.3.1) with the lme4 and lmerTest packages. Linear mixed-effects models (LMMs) were used to analyze longitudinal outcomes (PP-NRS, eGFR, and serum creatinine). Models included fixed effects for group (dupilumab vs. control), time (continuous, in days), and group × time interaction, with a patient-level random intercept to account for within-subject correlation. This approach utilizes all available observations under the missing-at-random (MAR) assumption via restricted maximum likelihood (REML) estimation, without requiring complete data at every time point. IGA was analyzed as a binary outcome (achievement of IGA 0/1 vs. IGA ≥ 2) reported descriptively due to sparse events. Serum creatinine was assessed for normality using the Shapiro–Wilk test and showed significant right-skewness (*P* < 0.05). Normally distributed continuous data are presented as mean ± standard deviation and compared using the Independent Samples *t*-test, while non-normally distributed data are expressed as median (IQR) and analyzed with the Mann–Whitney *U* test. Categorical variables are reported as frequency (percentage) and compared using the Chi-square or Fisher's exact test. A *p*-value <0.05 was considered statistically significant.

## 3 Results

### 3.1 Baseline characteristics

The mean age of the patients was 73.9 ± 10.9 years, with patients in dupilumab group (73.5 ± 10.59 years) slightly younger than those in control group (74.5 ± 12.32 years), but the difference was not statistically significant (*P* = 0.817). Male patients constituted the majority (26 cases, 86.7%).

Regarding comorbidities, hypertension had the highest prevalence (24 cases, 80.0% of the total), with a higher proportion in the control group (84.6%) than in the dupilumab group (76.5%), but the difference was not statistically significant (*P* = 0.672). Twelve patients (40.0%) had diabetes, with a higher proportion in the control group (53.8%) than in the dupilumab group (29.4%), also not statistically significant (*P* = 0.264). Ten patients (33.3%) had coronary heart disease, with a higher proportion in the control group (46.2%) than in the dupilumab group (23.5%). Although all *P* values were >0.05, the control group had higher proportions of hypertension, diabetes, and coronary heart disease than the dupilumab group, and this directional difference suggests possible residual confounding. Regarding atopic comorbidities, 5 patients (29.4%) in the dupilumab group and 2 patients (15.4%) in the control group had comorbid allergic rhinitis (*P* = 0.425). One patient (5.9%) in the dupilumab group had comorbid asthma; no patient in the control group had asthma (*P* = 1.000). No other atopic comorbidities (e.g., allergic conjunctivitis, food allergy) were documented in the medical records of either group.

The median AD duration was 11.5 months (IQR: 6.5–16.6) in the dupilumab group and 10.7 months (IQR: 5.0–13.4) in the control group, with no statistically significant difference (*P* = 0.568). The median CKD duration was 16.5 months (IQR: 9.5–41.5) in the dupilumab group, longer than the 11.9 months (IQR: 6.2–19.8) in the control group, also not statistically significant (*P* = 0.278). Overall, CKD duration was generally longer than AD duration in both groups, with the trend of shorter CKD duration in the control group consistent with their slightly better baseline renal function, reflecting the real distribution of different stages of renal failure progression. Among the 30 patients, 26 (86.7%) had a diagnosis of CKD that preceded or was concurrent with the diagnosis of AD, and only 4 patients (13.3%) had AD diagnosed prior to CKD. This indicates that CKD generally preceded or coexisted with AD in this cohort. This temporal pattern also highlights the need to distinguish AD from CKD-associated pruritus in this population.

Baseline disease activity assessment: The mean baseline IGA score was 3.97 ± 0.57, with no significant difference between groups (dupilumab group 3.94 ± 0.66 vs. control group 4.0 ± 0.43, *P* = 0.766). All enrolled patients met the inclusion criterion of baseline IGA ≥3 (range 3–5). The mean baseline PP-NRS score was 8.28 ± 0.84, indicating severe pruritus; the control group (8.58 ± 0.67) had a slightly higher score than the dupilumab group (8.06 ± 0.90), but the difference was not statistically significant (*P* = 0.080).

Regarding baseline treatment, 8 patients (26.7% of the total) were on low-dose oral corticosteroids (prednisone 5 mg/day), with no significant difference in the proportion of use between groups (dupilumab group 23.5% vs. control group 30.8%, *P* = 0.699). The proportion of topical corticosteroid use was high (19 cases, 63.3%). Most patients in the control group (12 cases, 92.3% of that group) used topical corticosteroid ointment, compared to 41.2% (7/17) in the dupilumab group, and the difference was statistically significant (*P* = 0.007). Oral second-generation antihistamines (cetirizine or loratadine, 10 mg once daily) were used by 16/17 (94.1%) patients in the dupilumab group and 13/13 (100%) patients in the control group (*P* = 1.000). In the control group, no patient received systemic immunosuppressive agents (methotrexate, mycophenolate mofetil, azathioprine, or cyclosporine) during the study period.

Baseline renal function indicators: The median (IQR) glomerular filtration rate (eGFR) was 20.41 (9.17–28.76) mL/min/1.73 m^2^, lower in the dupilumab group [18.05 (6.57–23.78)] than in the control group [20.53 (14.88–33.63)], but the difference was not statistically significant (*P* = 0.380). The median (IQR) serum Cr was 243 (174–508) μmol/L; the dupilumab group [258.0 (174.0–508.0)] was higher than the control group [224.5 (167.5–321.5)], also not statistically significant (*P* = 0.380).

In addition, for baseline Th2-type inflammatory biomarkers, the median serum total IgE levels were 173.0 (IQR: 41.38–382.75) IU/mL in the dupilumab group and 944.0 (IQR: 137.50–1,412.50) IU/mL in the control group (*P* = 0.157); the median peripheral blood eosinophil counts were 0.24 (IQR: 0.10–1.00) × 10^9^/L in the dupilumab group and 0.80 (IQR: 0.17–1.11) × 10^9^/L in the control group (*P* = 0.298). Neither difference was statistically significant.

### 3.2 Control of confounders

It should be noted that the proportion of topical corticosteroid use differed significantly between the two groups (*P* = 0.007), which might affect effectiveness observations. It is important to emphasize that the concomitant treatment data collected were limited to those retrievable from the hospital medical record system; out-of-hospital purchases by patients were not recorded. For patients with moderate-to-severe AD, the concomitant treatments including topical corticosteroids, TCIs, antihistamines, and emollients are all routine long-term basic treatments, so their impact on the observed effectiveness results is limited.

With regard to dialysis status, at baseline, 6 patients (35.3%) in the dupilumab group were already on maintenance dialysis (5 on maintenance hemodialysis, 1 on peritoneal dialysis), while none of the patients in the control group were on dialysis at baseline (0%), and the difference between the two groups was statistically significant (*P* = 0.024). It must be pointed out that given the small sample size of this study, this baseline difference may involve some selection bias. To avoid the direct physical intervention of dialysis on serum creatinine clearance and to eliminate the potential interference of this bias on the results, we performed a sensitivity stratified analysis in the subsequent renal safety analysis. Detailed dialysis adequacy parameters and frequency-adjusted dialysis measures were not consistently available in the retrospective records, which may have influenced the interpretation of serum creatinine and eGFR trajectories.

Regarding baseline CKD treatment, most patients received therapies aimed at improving renal hemodynamics or protecting renal function. Specifically, 15 patients (88.2%) in the dupilumab group and 10 patients (76.9%) in the control group received renin-angiotensin-aldosterone system (RAAS) inhibitors, such as ACEIs/ARBs, or diuretics, such as furosemide, torasemide. The remaining patients received other renoprotective treatments according to nephrology practice.

All enrolled CKD stage 4–5 patients were managed by the nephrology department and received professional dietary counseling, including low-protein dietary recommendations. However, individual dietary adherence and actual protein intake could not be quantified retrospectively.

In terms of safety assessment (including injection site reactions, conjunctivitis, and herpes simplex virus infections), none of these adverse reactions were recorded in the clinical records of any patient, and no treatment was interrupted due to adverse events.

### 3.3 Effectiveness analysis

At baseline (day 0), the control group had a mean IGA of 4.0 and the dupilumab group had a mean IGA of approximately 3.88. The control group had a mean PP-NRS of 8.7, and the dupilumab group had a mean PP-NRS of approximately 8.0, indicating similar baseline severity with no significant differences.

At the Day 30 follow-up: the control group mean IGA was 2.4, a decrease of 1.6 points from baseline (within-group *P* < 0.001); the dupilumab group mean IGA was 2.4, a decrease of approximately 1.48 points (within-group *P* < 0.001); the between-group difference in IGA was not statistically significant (*P* = 0.865, 95% CI: −0.79 to 0.92). The control group mean PP-NRS was 6.0, a decrease of 2.7 points (within-group *P* < 0.001); the dupilumab group mean PP-NRS was 3.4, a decrease of approximately 4.6 points (within-group *P* < 0.001); the between-group difference was not statistically significant (*P* = 0.128, 95% CI: −3.07 to 0.29), but both groups showed early improvement.

At Day 60: the control group mean IGA was 3.0, the dupilumab group mean IGA was 3.0; the between-group difference was not significant (*P* = 0.721, 95% CI: −2.34 to 1.68). The control group mean PP-NRS was 6.0, the dupilumab group mean PP-NRS was 5.0; the between-group difference was also not statistically significant (*P* = 0.593, 95% CI: −5.97 to 3.47).

At Day 90: the control group mean IGA was 2.67, the dupilumab group mean IGA was 2.5 (between-group *P* = 1.000, 95% CI: −2.07 to 2.07). The control group mean PP-NRS was 5.0, the dupilumab group mean PP-NRS was 3.67 (between-group *P* = 0.374, Cohen's d = −0.82). Although the between-group difference was not statistically significant, the large effect size (Cohen's *d* = 0.82) suggests that the dupilumab group had a more pronounced numerical reduction in pruritus.

**Figure 1:**
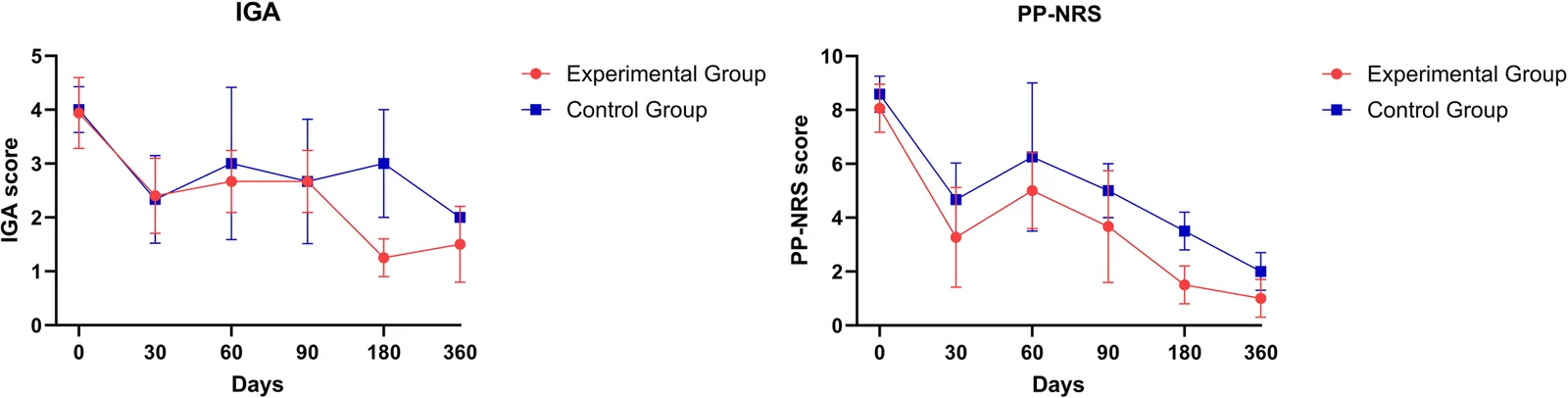
Changes in disease severity scores in patients with AD at each visit after dupilumab treatment. IGA, investigator's global assessment; PP-NRS, peak pruritus numeric rating scale.

Overall (Figure 1), the linear mixed-effects model for PP-NRS showed a highly significant effect of time (*β* = −0.018/day, *P* < 0.001), a trend toward lower overall scores in the dupilumab group that did not reach significance (*β* = −1.19, *P* = 0.109), and a non-significant group × time interaction (*β* = −0.002, *P* = 0.796). For IGA analyzed as a continuous variable, neither the group effect (*P* = 0.996), time effect (*P* = 0.074), nor the interaction (*P* = 0.749) reached statistical significance. During follow-up, 4 patients (13.3%) achieved IGA 0/1, with no significant difference between groups (dupilumab 11.8% vs. control 15.4%, *P* = 1.000). These results indicate that while both groups improved significantly over time, the dupilumab group showed a numerical trend toward greater PP-NRS reduction that did not achieve statistical significance after accounting for within-subject correlation. This finding should be interpreted as suggestive of a potential benefit rather than confirmatory of therapeutic superiority (see Supplementary Table S2 for full model results).

### 3.4 Renal safety analysis

At the 30-day follow-up, the dupilumab group's mean eGFR decreased by 0.9 mL/min/1.73 m^2^ from baseline, and mean Cr decreased by 63.80 umol/L; the control group's mean eGFR decreased by 1.95 mL/min/1.73 m^2^, and Cr increased by 38.9 umol/L.

At the 60-day follow-up, the dupilumab group's eGFR decreased by 3.06 mL/min/1.73 m^2^ from baseline, and mean Cr increased by 144.67umol/L; the control group's eGFR decreased by 0.41 mL/min/1.73 m^2^, and Cr decreased by 90.50 umol/L.

At the 90-day follow-up, the dupilumab group's eGFR decreased by 4.63 mL/min/1.73 m^2^ from baseline, and mean Cr increased by 128.7 umol/L; the control group's eGFR decreased by 0.88 mL/min/1.73 m^2^, and Cr decreased by 87.0 umol/L.

At the 180-day follow-up, the dupilumab group's eGFR decreased by 0.97 mL/min/1.73 m^2^ from baseline, and Cr increased by 59.45 umol/L from baseline; the control group's eGFR decreased by 4.91 mL/min/1.73 m^2^, and Cr decreased by 35.79 umol/L.

**Figure 2:**
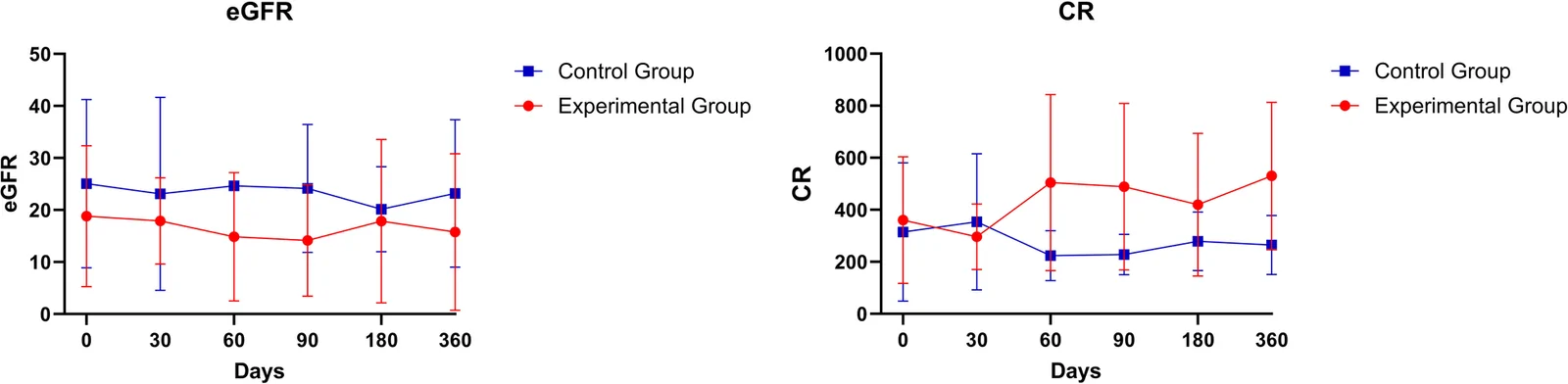
Renal function changes in patients with AD at each visit after dupilumab treatment. eGFR, estimated Glomerular Filtration Rate; CR, Creatinine.

At the 1-year follow-up, the dupilumab group's mean eGFR decreased by 3.04 mL/min/1.73 m^2^ from baseline, and Cr increased by 170.43 μmol/L; the control group's mean eGFR decreased by 1.87 mL/min/1.73 m², and Cr decreased by 49.90 μmol/L. No statistically significant differences were found between the two groups at day 360 for eGFR (*P* = 0.374) or Cr (*P* = 0.077). Despite worse baseline renal function, the dupilumab group did not show sustained deterioration of renal function; eGFR returned to levels close to the control group at 180 days, and the overall change trend was consistent with the control group (Figure 2).

Linear mixed-effects models for eGFR showed non-significant effects for group (*β* = −5.08, *P* = 0.386), time (*β* = −0.016/day, *P* = 0.182), and group  ×  time interaction (*β* = 0.015, *P* = 0.279). For serum creatinine, the group effect (*β* = 130.30, *P* = 0.120), time effect (*β* = 0.178/day, *P* = 0.147), and interaction (*β* = −0.133, *P* = 0.402) were similarly non-significant (Supplementary Table S2). The non-significant interaction indicates that renal function trajectories did not differ detectably between groups. However, given the small sample size and limited statistical power, the absence of a significant difference should not be interpreted as proof of renal safety, and the possibility of a clinically meaningful difference that this study was underpowered to detect (type II error) cannot be excluded. To further control for the interference of dialysis on renal function (eGFR and Cr) trajectories, we performed a sensitivity analysis by removing the 6 patients on dialysis in the experimental group (5 hemodialysis, 1 peritoneal dialysis) and comparing the two groups of completely non-dialyzed patients (dupilumab group *n* = 11, control group *n* = 13). After excluding dialysis patients, there were no significant differences between the dupilumab group and the control group in baseline eGFR (*P* = 0.246) or Cr (*P* = 0.395). At the 180-day follow-up, the between-group difference in eGFR was not statistically significant (*P* = 0.496), and the between-group difference in Cr was also not significant (*P* = 0.606). At the 1-year follow-up (day 360), eGFR (*P* = 0.287) and Cr (*P* = 0.480) remained not statistically significantly different between the dupilumab group and the control group.

## 4 Discussion

This study is the first to focus on the application of dupilumab in AD patients with severe renal failure. The results suggest a numerical trend toward greater pruritus reduction in the dupilumab group compared with controls, though between-group differences did not reach statistical significance in the mixed-effects analysis (PP-NRS group effect: *β* = −1.19, *P* = 0.109). No statistically detectable difference in renal function trajectories was observed between groups. In the sensitivity analysis excluding dialysis patients, there were no statistically significant differences in eGFR or serum creatinine trajectories between groups during the 1-year follow-up, further supporting the absence of a statistically detectable acceleration of renal function decline, though these findings remain preliminary and do not constitute definitive confirmation of renal safety.

In the pharmacokinetics (PK) of dupilumab after subcutaneous (SC) administration, peak plasma drug concentrations are reached 3–7 days after injection, and Cmax is similar to the dose ratio, indicating that plasma drug concentrations increase proportionally with the administered dose (4). The SC bioavailability of dupilumab is approximately 61%–64%. Due to its large molecular size (147 kDa), Dupilumab's distribution *in vivo* is primarily confined to the systemic circulation, and its metabolism/elimination pathways have not been characterized. Since dupilumab is a fully human monoclonal IgG4 antibody, its primary elimination pathway is expected to involve intracellular proteolytic degradation into small peptides and amino acids within tissue cells, similar to the catabolic fate of endogenous IgG. The primary sites of metabolism for large proteins such as monoclonal antibodies are the liver, blood, and extravascular tissue sites; biologics are not excreted unchanged in urine or feces. This non-renal elimination mechanism suggests that renal impairment would not significantly affect dupilumab clearance (1, 5). This characteristic potentially reduces the risk of drug accumulation in patients with renal insufficiency, consistent with the safety results observed in this study. Furthermore, given its large molecular weight (147 kDa), dupilumab far exceeds the molecular weight cutoff of standard hemodialysis membranes (approximately 15–20 kDa) and even high-flux dialyzers (typically ≤40–50 kDa). Therefore, dupilumab cannot be meaningfully filtered or removed by either conventional hemodialysis or peritoneal dialysis, and no dosage adjustment is required for patients on maintenance dialysis (5). Another study reviewed 18 patients with both AD and Chronic Renal Insufficiency (CRI). After receiving dupilumab, the patients' disease severity and quality of life improved significantly. No renal function deterioration occurred. This confirms the drug's safety and effectiveness (6). However, this study did not strictly distinguish between patients with moderate to severe renal failure and did not include a control group. Natural renal function changes in AD patients with renal failure who did not take dupilumab were not analyzed or compared. Furthermore, A phase III trials of dupilumab showed a favorable and consistent safety profile for dupilumab alone or in combination with topical Corticosteroids (TCS) in the treatment of patients with moderate-to-severe AD (7).

Dupilumab inhibits IL-4/IL-13 signaling by blocking IL-4Rα, which not only improves skin lesions but may also reduce systemic inflammation. AD patients often have systemic Th2 inflammation (such as eosinophil activation, elevated IL-5), and chronic inflammation has been shown to accelerate microangiopathy and oxidative stress, indirectly damaging renal function (8). There is evidence that serum interleukin (IL)-31 is elevated in patients with uremic (CKD stage 5/ ≤ 15 mL/min) pruritus (CKD-aP) (9). Dupilumab may indirectly reduce pruritus by suppressing Th2 cell production of IL-31, a key pruritogenic cytokine whose expression is driven by IL-4/IL-13 signaling (10, 11). A study on the use of dupilumab for chronic kidney disease-associated pruritus in hemodialysis patients showed that dupilumab significantly reduced pruritus in hemodialysis patients with CKD-aP (12). In that report, CKD-aP patients experienced significant relief of pruritus after dupilumab treatment and achieved an 83.3% EASI-90 response rate. This also suggests that the slight rebound in eGFR observed in the dupilumab group of this study might be related to improved renal microenvironment after inflammation control, but this requires further validation. Of note, an apparent divergence was observed between pruritus improvement and IGA response in this cohort. While the dupilumab group showed a numerical trend toward greater PP-NRS reduction, IGA scores did not differ significantly between groups. This divergent pattern may be explained by the multifactorial nature of pruritus and skin morphology in CKD patients. In patients with CKD stages 4–5, pruritus is driven by both AD-related Th2 inflammation and CKD-associated pruritic pathways (uremic toxins, calcium-phosphate imbalance, systemic inflammation, and IL-31 elevation). Dupilumab, by blocking IL-4/IL-13 signaling, may effectively suppress the Th2-driven pruritogenic component, resulting in substantial itch relief. However, IGA reflects the overall morphological severity of skin lesions, which in CKD patients may be influenced by additional factors beyond Th2 inflammation—including uremic xerosis from altered skin barrier, excoriation-related secondary changes, and medication-related skin alterations—that are not directly targeted by dupilumab. It should be noted that following the revised linear mixed-effects analysis, the group effect for PP-NRS no longer reached statistical significance (*P* = 0.109), and we have accordingly revised our conclusions to describe this as a numerical trend rather than demonstrated superiority.

Overall, the dupilumab group demonstrated a numerical trend toward greater pruritus improvement that is consistent with the drug's known pharmacological activity, though the small sample size and non-significant between-group comparisons preclude definitive conclusions regarding superiority over conventional therapy. A reported case of an AD patient with severe renal failure using dupilumab showed significant improvement in AD symptoms and no observed drug-related renal function deterioration (13). Another report described a patient with chronic kidney disease (CKD) and severe AD who could not use traditional systemic therapy (such as cyclosporine) due to poor renal function. After switching to dupilumab, the AD was controlled, and no renal function deterioration occurred (2-year follow-up) (14).

The present study has several limitations. First, the small sample size and lack of an *a priori* sample size calculation limited the statistical power to detect small or moderate differences in renal outcomes, resulting in a risk of type II error when interpreting renal safety. Second, the retrospective single-center design inevitably introduces selection bias and residual confounding. Treatment assignment was not randomized but was based on real-world clinical decision-making, including disease refractoriness, physician–patient discussion, treatment accessibility, and patients' economic considerations. Therefore, the observed associations should not be interpreted as definitive causal effects. The follow-up time is relatively short, precluding assessment of potential long-term effects of the drug on renal function. In patients with advanced CKD, AD-related pruritus and CKD-associated pruritus may overlap clinically and pathophysiologically. Both conditions may involve systemic inflammation and IL-31-related pruritic pathways (15). Although we excluded patients with isolated generalized pruritus without primary inflammatory eczematous lesions, the uremic microenvironment may still have aggravated pruritus burden and complicated phenotype differentiation. This represents an important diagnostic and analytical limitation of the present study, particularly when interpreting PP-NRS improvement. In addition, the EASI score is an important standard for objective severity assessment in AD clinical trials; in routine clinical practice, EASI scores were not systematically recorded in patients' electronic medical records and could not be extracted retrospectively. We used IGA and PP-NRS as endpoint measures, which were completely recorded. This is an important limitation. Finally, because routine clinical practice does not mandate repeated laboratory tests for patients, although we collected baseline Th2 biomarker data (such as total IgE, peripheral blood eosinophil counts), we still lack Th2 biomarker data during treatment and follow-up, which also limits our ability to track their changes over time. Lastly, all patients in this study came from a single tertiary hospital in Wuhan, China; their demographic characteristics and healthcare environment may differ from those of other regions or countries, so caution is needed when extrapolating the conclusions to broader populations or different healthcare systems.

## 5 Conclusion

In this real-world retrospective cohort of AD patients with CKD stages 4–5, dupilumab was associated with a numerical trend toward greater improvement in pruritus compared with conventional therapy, and was not associated with a statistically detectable acceleration of renal function decline. However, given the small sample size, retrospective design, baseline imbalance, and limited follow-up, these findings should be considered preliminary. Larger prospective studies are needed to further evaluate the effectiveness and renal safety of dupilumab in this high-risk population.

## Data Availability

The raw data supporting the conclusions of this article will be made available by the authors, without undue reservation.

## References

[1] McCann MR Kosloski MP Xu C Davis JD Kamal MA. Dupilumab: mechanism of action, clinical, and translational science. Clin Transl Sci. (2024) 17(8):e13899. 10.1111/cts.13899 39080841 PMC11288895

[2] Chen TK Knicely DH Grams ME. Chronic kidney disease diagnosis and management: a review. JAMA. (2019) 322(13):1294–304. 10.1001/jama.2019.14745 31573641 PMC7015670

[3] Chu DK Schneider L Asiniwasis RN Boguniewicz M De Benedetto A Ellison K. Atopic dermatitis (eczema) guidelines: 2023 American academy of allergy, asthma and immunology/American college of allergy, asthma and immunology joint task force on practice parameters GRADE- and institute of medicine-based recommendations. Ann Allergy Asthma Immunol. (2024) 132(3):274–312. 10.1016/j.anai.2023.11.009 38108679

[4] Li Z Radin A Li M Hamilton JD Kajiwara M Davis JD. Pharmacokinetics, pharmacodynamics, safety, and tolerability of dupilumab in healthy adult subjects. Clin Pharmacol Drug Dev. (2020) 9(6):742–55. 10.1002/cpdd.798 32348036 PMC7496261

[5] Baumann A. Preclinical development of therapeutic biologics. Expert Opin Drug Discov. (2008) 3(3):289–97. 10.1517/17460441.3.3.289 23480264

[6] Peng C Cao Q Xiong F Xu H Li J. Effectiveness and safety of dupilumab in moderate-to-severe atopic dermatitis patients with chronic renal insufficiency: a real-world retrospective study in China. MedComm. (2024) 5(10):e707. 10.1002/mco2.707 39309693 PMC11413493

[7] Gooderham MJ Hong HC Eshtiaghi P Papp KA. Dupilumab: a review of its use in the treatment of atopic dermatitis. J Am Acad Dermatol. (2018) 78(3 Suppl 1):S28–36. 10.1016/j.jaad.2017.12.022 29471919

[8] Wang S Zeng J Chen Y Sun F Ma H Zhang L. Association between systemic inflammation and worsening renal function in cardiovascular-kidney-metabolic syndrome. Am J Nephrol. (2025) 56(6):751–61. 10.1159/000546130 40294586

[9] Swierczynska K Krajewski PK Nowicka-Suszko D Bialynicki-Birula R Krajewska M Szepietowski JC. The serum level of IL-31 in patients with chronic kidney disease-associated pruritus: What Can We Expect? Toxins (Basel). (2022) 14(3): 197. 10.3390/toxins14030197 35324695 PMC8955714

[10] Guttman-Yassky E Bissonnette R Ungar B Suárez-Fariñas M Ardeleanu M Esaki H. Dupilumab progressively improves systemic and cutaneous abnormalities in patients with atopic dermatitis. J Allergy Clin Immunol. (2019) 143(1):155–72. 10.1016/j.jaci.2018.08.022 30194992

[11] Kabashima K Irie H. Interleukin-31 as a clinical target for pruritus treatment. Front Med (Lausanne). (2021) 8:638325. 10.3389/fmed.2021.638325 33644103 PMC7906974

[12] Wang Z Chen C Xiong Y Feng Y Chen J Xu H. Efficacy of dupilumab for chronic kidney disease-associated pruritus in patients undergoing hemodialysis: retrospective observational study. J Am Acad Dermatol. (2025) 93(2):501–3. 10.1016/j.jaad.2025.03.067 40158547

[13] Foti C Romita P Ambrogio F Manno C Filotico R Cassano N. Treatment of severe atopic dermatitis with dupilumab in three patients with renal diseases. Life (Basel). (2022) 12(12):2002. 10.3390/life12122002 36556367 PMC9785101

[14] Phelps-Polirer K Alkhatib BL Davis C. Generalized granuloma Annulare associated with dupilumab therapy. Cureus. (2022) 14(7):e27439. 10.7759/cureus.27439 36051735 PMC9420399

[15] Makar M Smyth B Brennan F. Chronic kidney disease-associated pruritus: a review. Kidney and Blood Pressure Research. (2021) 46(6):659–69. 10.1159/000518391 34515143

