# Supplementary material for: Effectiveness and renal safety of dupilumab in the treatment of moderate-to-severe atopic dermatitis patients with chronic kidney disease stages 4–5: a real-world cohort study in China
Source: Front Med (Lausanne). 2026 Aug 27;13:1889589. doi: 10.3389/fmed.2026.1889589 (PMC13557936; doi:10.3389/fmed.2026.1889589)
Supplement: Supplementary file 1 [file Supplementaryfile1.docx]

**Table S1. Number of Patients with Available Data at Each Time Point**

| **Time Point** | **Dupilumab (n=17)** | | | **Control (n=13)** | | |
| --- | --- | --- | --- | --- | --- | --- |
|  | PP-NRS | IGA | eGFR/Cr | PP-NRS | IGA | eGFR/Cr |
| Day 0 | 17 | 17 | 15 | 13 | 13 | 10 |
| Day 30 | 10 | 10 | 9 | 9 | 9 | 7 |
| Day 60 | 2 | 3 | 6 | 4 | 4 | 5 |
| Day 90 | 3 | 4 | 8 | 4 | 2 | 3 |
| Day 180 | 2 | 3 | 10 | 2 | 1 | 5 |
| Day 360 | 2 | 2 | 7 | 2 | 0 | 4 |
