# Supplementary material for: Effectiveness and renal safety of dupilumab in the treatment of moderate-to-severe atopic dermatitis patients with chronic kidney disease stages 4–5: a real-world cohort study in China
Source: Front Med (Lausanne). 2026 Aug 27;13:1889589. doi: 10.3389/fmed.2026.1889589 (PMC13557936; doi:10.3389/fmed.2026.1889589)
Supplement: Supplementary file 2 [file Supplementaryfile2.docx]

**Supplementary Table S2. Linear Mixed-Effects Model Results for Longitudinal Outcomes**

| **Outcome** | **Fixed Effect** | **β Estimate** | **95% CI** | **P-value** |
| --- | --- | --- | --- | --- |
| **PP-NRS** | Intercept | 8.14 | (7.31, 8.97) | <0.001 |
|  | Group (Dupilumab vs Control) | −1.19 | (−2.66, 0.28) | 0.109 |
|  | Time (per day) | −0.018 | (−0.024, −0.012) | <0.001 |
|  | Group × Time | −0.002 | (−0.011, 0.007) | 0.796 |
| **IGA** | Intercept | 3.89 | (3.42, 4.36) | <0.001 |
|  | Group (Dupilumab vs Control) | 0.002 | (−0.65, 0.66) | 0.996 |
|  | Time (per day) | −0.010 | (−0.020, 0.001) | 0.074 |
|  | Group × Time | 0.002 | (−0.008, 0.011) | 0.749 |
| **eGFR** (mL/min/1.73m²) | Intercept | 25.12 | (15.83, 34.41) | <0.001 |
|  | Group (Dupilumab vs Control) | −5.08 | (−16.72, 6.56) | 0.386 |
|  | Time (per day) | −0.016 | (−0.040, 0.008) | 0.182 |
|  | Group × Time | 0.015 | (−0.012, 0.042) | 0.279 |
| **Serum Creatinine** (μmol/L) | Intercept | 241.50 | (131.20, 351.80) | <0.001 |
|  | Group (Dupilumab vs Control) | 130.30 | (−34.50, 295.10) | 0.120 |
|  | Time (per day) | 0.178 | (−0.064, 0.420) | 0.147 |
|  | Group × Time | −0.133 | (−0.447, 0.181) | 0.402 |
